# Risk factors of secondary cancer in nasopharyngeal carcinoma patients after radiotherapy

**DOI:** 10.7150/jca.77768

**Published:** 2022-10-09

**Authors:** Wen-Jie Wang, Miao Li, Xin-Bin Pan

**Affiliations:** 1 Department of Oncology, The Central Hospital of Wuhan, Tongji Medical College, Huazhong University of Science and Technology, Wuhan, Hubei 430014, P.R. China.; 2 Department of Radiation Oncology, Guangxi Medical University Cancer Hospital, Nanning, Guangxi 530021, P.R. China.

**Keywords:** nasopharyngeal carcinoma, secondary cancer, risk factor, nomogram

## Abstract

**Purpose:** To identify risk factors of secondary cancer in nasopharyngeal carcinoma (NPC) patients after radiotherapy.

**Materials and methods:** The data of NPC patients with secondary cancer were extracted from the Surveillance, Epidemiology, and End Results database from 2004 to 2016. Univariate and multivariate logistic regression analysis was performed to identify risk factors of secondary cancer. Risk factors selected from the multivariable logistic regression analysis were used to build a predicting model.

**Results:** A total of 3931 patients were included: 329 (8.37%) patients developed secondary cancers and 3602 (91.63%) patients did not have secondary cancers. Univariate logistic regression analysis revealed that age, race, and the American Joint Committee on Cancer (AJCC) stage were risk factors of secondary cancer. Multivariable analysis demonstrated that age [Odds ratio (OR) = 1.03, P < 0.001], race (OR = 1.17, P = 0.010), AJCC stage (OR = 0.82, P = 0.002), and chemotherapy (OR = 1.55, P = 0.028) were independent risk factors of secondary cancer. Age, race, AJCC stage, and chemotherapy were entered into a nomogram for predicting secondary cancer. The area under the ROC curve of the nomogram was 0.645 [95% confidence interval (CI): 0.617-0.673]. The decision curve showed that if the threshold probability is between 4% and 25%, using the nomogram added more benefit than either the treat-all-patients scheme or the treat-none scheme.

**Conclusion:** Age, race, AJCC stage, and chemotherapy were independent risk factors of secondary cancer in nasopharyngeal carcinoma patients after radiotherapy.

## Introduction

Nasopharyngeal carcinoma (NPC) is a highly epidemiologic and radiosensitive cancer 1, 2. Radiotherapy is the primary treatment for NPC 3, 4. With the improvement in diagnosis and treatment, long-term survival of NPC patients are increasing. As a result, secondary cancer after radiotherapy becomes a serious complication among these long-term survivors 5, 6. Although the secondary cancer is rare 7-10, it can decrease patients' survivals 11. The low frequency of secondary cancer makes it difficult to identify the potential predictive factors. This retrospective study was conducted to identify risk factors of secondary cancer in NPC patients after radiotherapy using the data of the Surveillance, Epidemiology, and End Results (SEER) database.

## Materials and Methods

### Data source and patients

This retrospective study searched the SEER database to extract data of NPC patients from 2004 to 2016. The inclusion criteria were as follows. (1) Pathologically confirmed NPC. (2) definite TNM stages of the American Joint Committee on Cancer (AJCC), (3) NPC was the first cancer, (4) not stage M1, and (5) received radiotherapy. Patients' characteristics of age, sex, race, tumor grade, World Health Organization (WHO) classification, AJCC stage, chemotherapy, and secondary cancer were extracted.

### Identifying risk factors

Univariate logistic regression analysis was performed to identify potential risk factors of secondary cancer. All the patients' characteristics of age, sex, race, tumor grade, WHO classification, AJCC stage, and chemotherapy were included in the univariate logistic regression analysis. All the factors were also included in the multivariate logistic regression analysis to identify independent risk factors. Factors with a *P* < 0.05 in the multivariable logistic regression analysis were considered as the independent risk factors of secondary cancer. The results of logistic regression analysis were reported as Odds ratios (ORs) with 95% confidence intervals (CIs).

### Nomogram development

The independent risk factors of secondary cancer identified from the multivariable logistic regression analysis were used to develop a predictive nomogram. Receiver operating characteristic (ROC) curve analysis was used to assess the nomogram discrimination capacity. The area under the ROC curve (AUC) was calculated for quantification. The performance of the nomogram was assessed by a calibration plot for internal calibration. The decision curve analysis (DCA) was adopted to evaluate the clinical efficacy of the nomogram and analyze the net benefit under different risk thresholds.

### Statistical analysis

The continuous variable of age was compared using Wilcoxon rank sum test between secondary cancer group and non-secondary cancer group. Categorical variables, including sex, race, tumor grade, WHO classification, AJCC stage, and chemotherapy, were analyzed using the χ^2^ test or Fisher's exact test. Overall survival between the secondary cancer group and non-secondary cancer group were calculated using the Kaplan-Meier analysis with log-rank test statistics. Multivariable proportional hazards models adjusted for age, sex, race, tumor grade, WHO classification, AJCC stage, and chemotherapy were implemented to assess independent prognostic factors.

Statistical analyses were performed using SPSS Statistics Version 26.0 software (IBM Co., Armonk, NY, USA) and R software (version 4.0.2). Two-tailed *P* values < 0.05 were considered statistically significant.

## Results

### Patient characteristics

**Figure 1:**
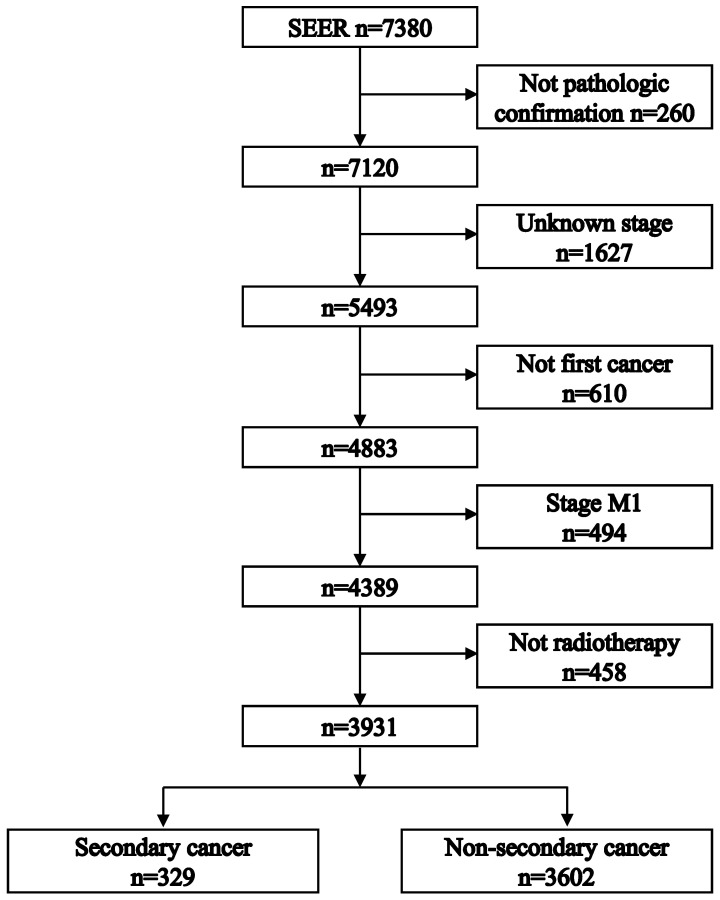
Flowchart depicting patient selection.

Figure 1 shows the process of patient selection. A total of 3931 patients were included. The secondary cancer group included 329 (8.37%) patients. The non-secondary cancer group included 3602 (91.63%) patients. Table 1 summarizes the patient characteristics. The median follow-up times were 46 [interquartile range (IQR): 20-85] months in the non-secondary cancer group and 69 (IQR: 39-102) months in the secondary cancer group, respectively.

### Survival between the secondary cancer and non-secondary cancer groups

**Figure 2:**
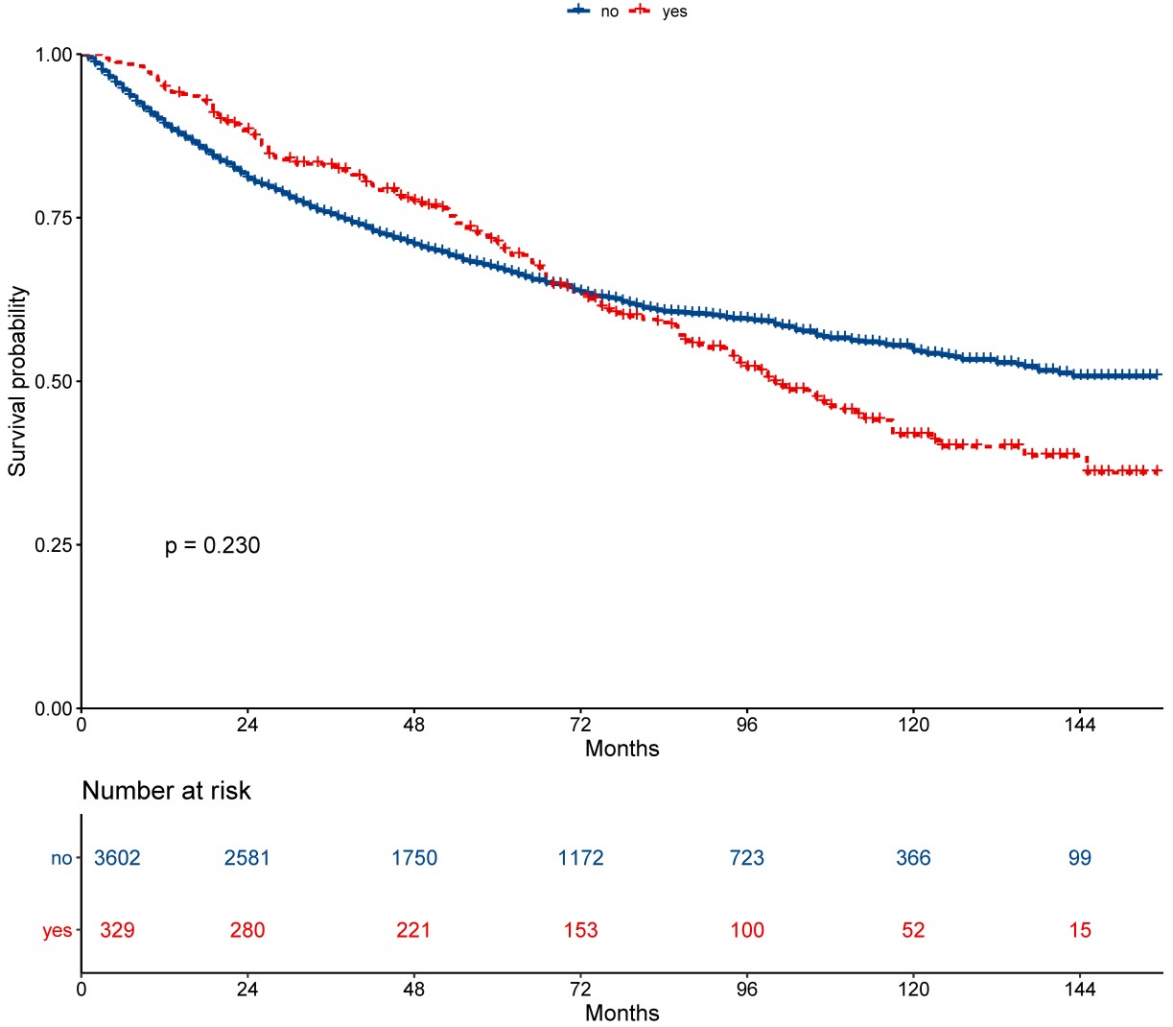
Survival between patients with secondary cancer and without secondary cancer.

**Figure 3:**
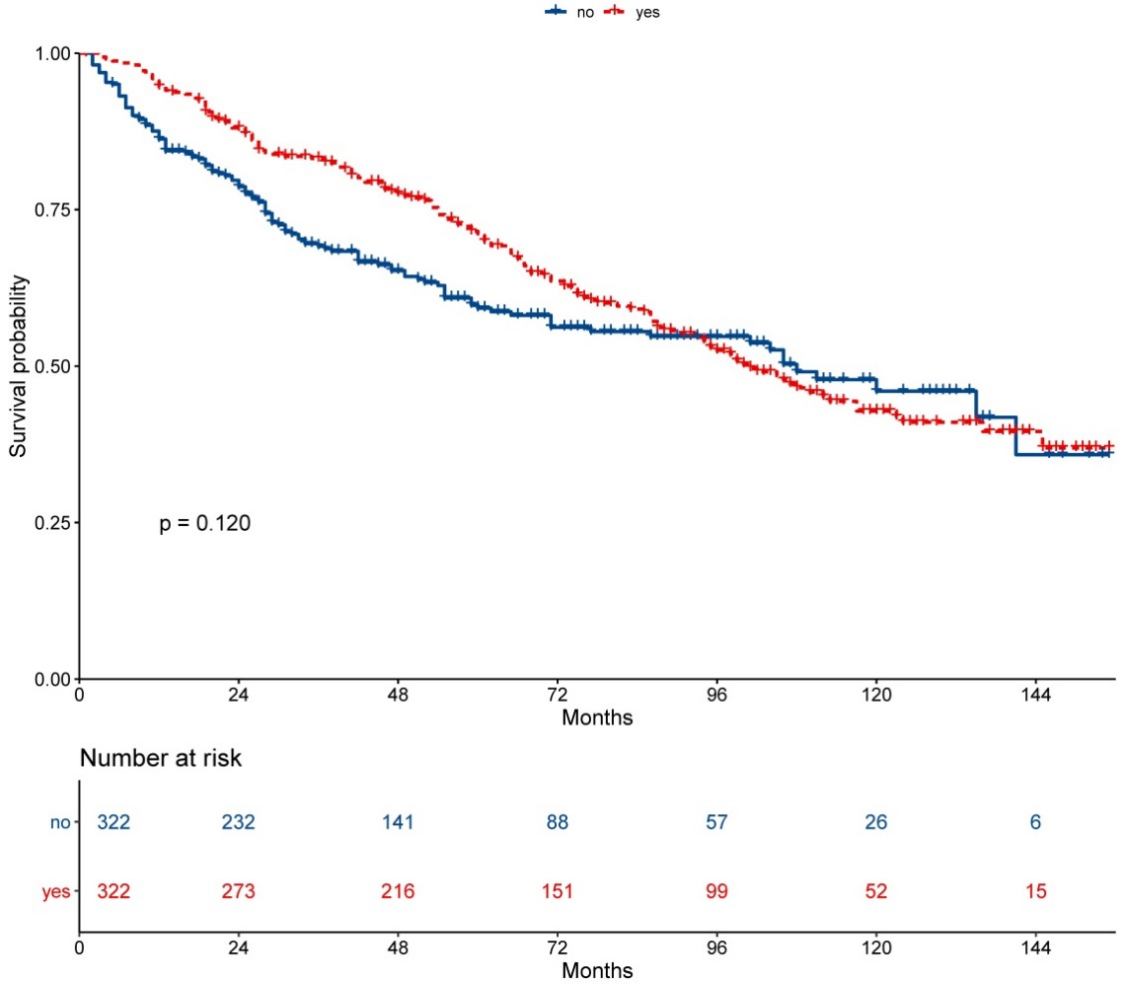
Survival between patients with secondary cancer and without secondary cancer after propensity score matching.

The 5-year overall survival did not differ between the secondary cancer and non-secondary cancer groups (71.2% vs. 67.2%; *P* = 0.230, Figure 2). The 7-year overall survival of the secondary cancer and non-secondary cancer groups was 63.4% and 63.6%. Overall survival of the secondary cancer group was worse than that of the non-secondary cancer group in 7^th^ year after radiotherapy. Patient characteristics after propensity score matching were showed in Table 2. After propensity score matching, the 5-year overall survival was similar between the secondary cancer and non-secondary cancer groups (59.3% vs. 71.1%; *P* = 0.120, Figure 3).

**Figure 4:**
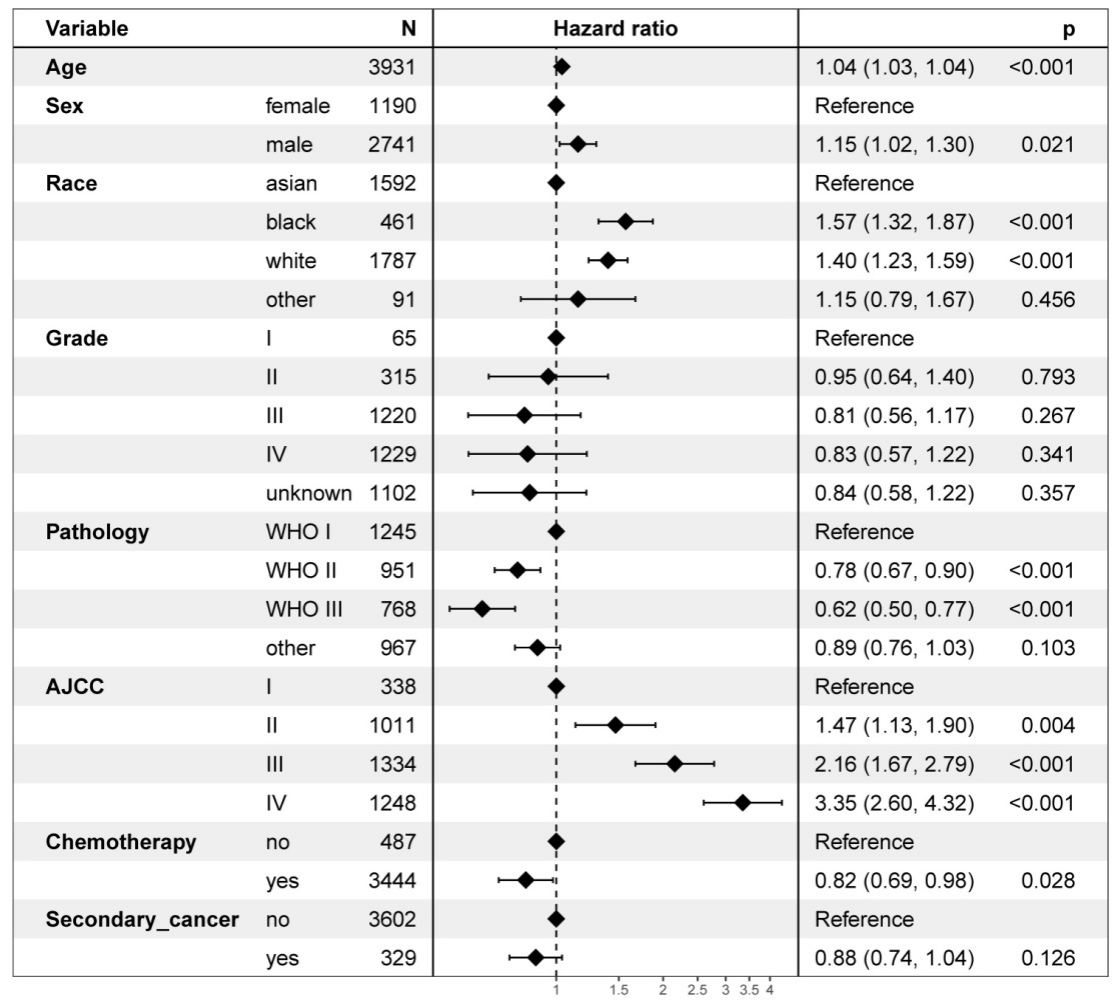
Multivariate regression analysis of prognostic factors.

**Figure 5:**
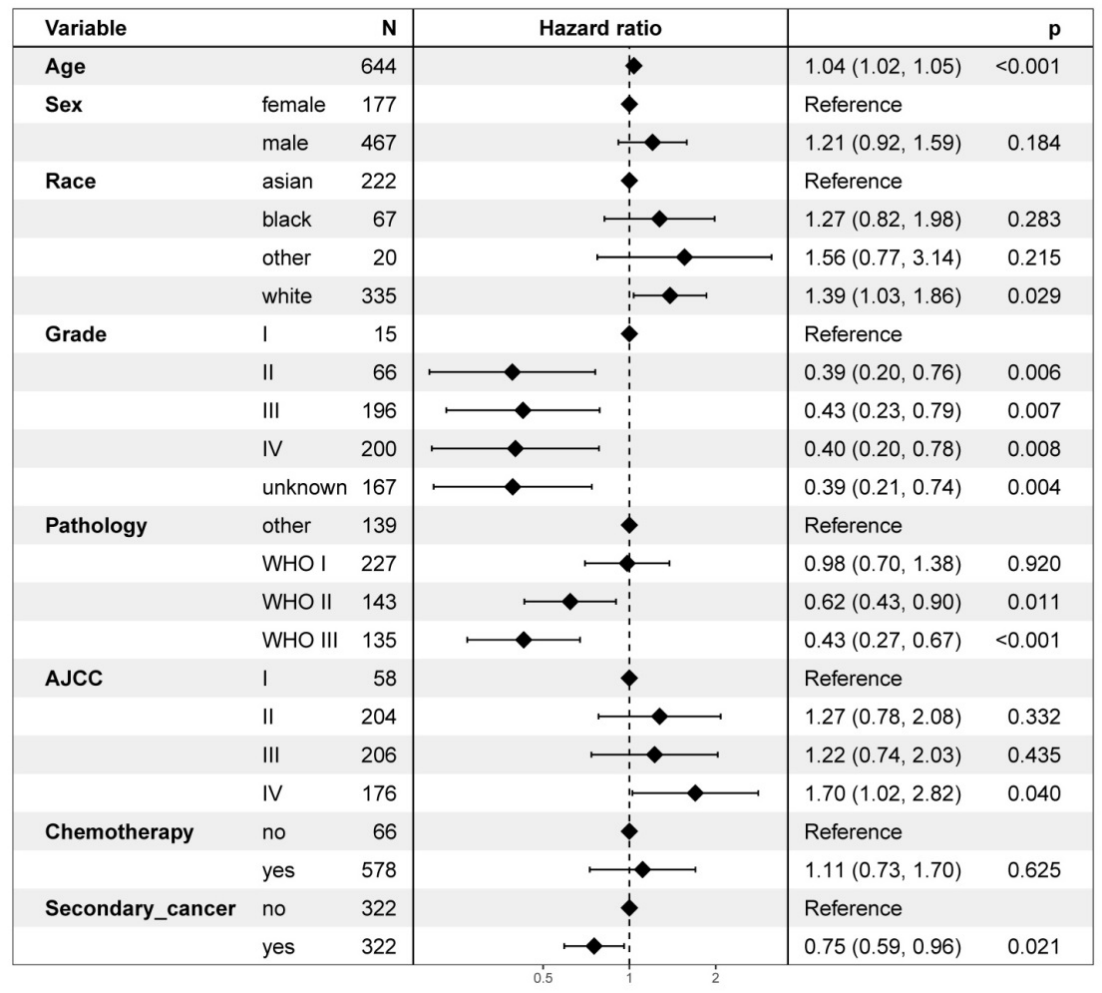
Multivariate regression analysis of prognostic factors after propensity score matching.

On multivariable proportional hazards model, factors of age, sex, race, WHO classification, AJCC stage, and chemotherapy were independent prognostic factors for OS. However, secondary cancer was not an independent prognostic factor for OS (hazard ratio = 0.88, 95% CI: 0.74-1.04; *P* = 0.126, Figure 4). After propensity score matching, secondary cancer was an independent prognostic factor for OS (hazard ratio = 0.75, 95% CI: 0.59-0.96; *P* = 0.021, Figure 5).

### Independent risk factors of secondary cancer

**Figure 6:**
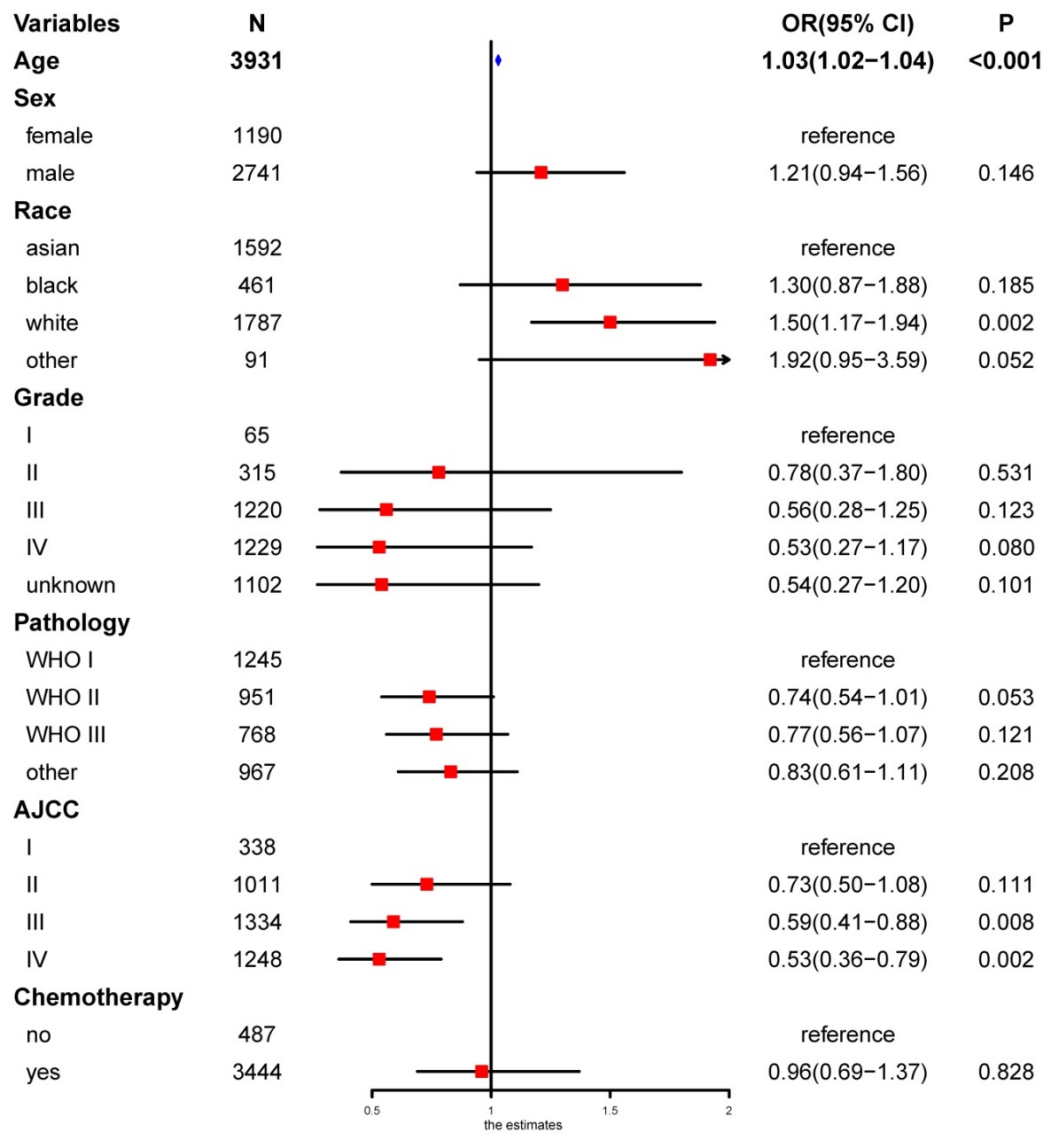
Univariate logistic regression analysis for risk factors of secondary cancer.

**Figure 7:**
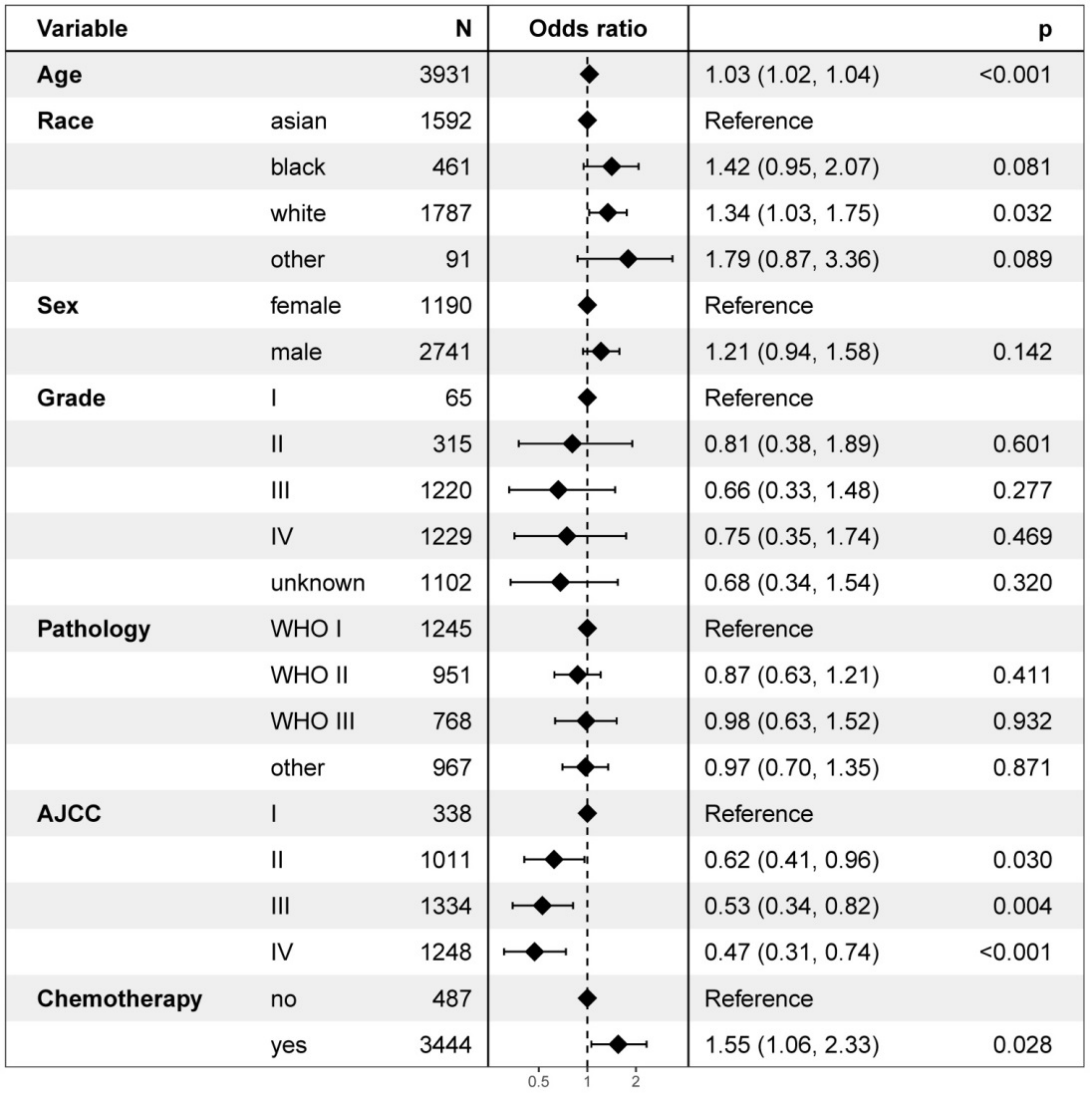
Multivariable logistic regression analysis for independent risk factors of secondary cancer.

Univariate logistic regression analysis revealed that age, race, and the AJCC stage were risk factors of secondary cancer (Figure 6). Multivariable logistic regression analysis demonstrated that age (OR = 1.03, *P* < 0.001), race (OR = 1.17, *P* = 0.010), AJCC stage (OR = 0.82, *P* = 0.002), and chemotherapy (OR = 1.55, *P* = 0.028) were independent risk factors of secondary cancer (Figure 7). Chemotherapy was not a risk factor of secondary cancer in the univariate logistic regression analysis. However, it was an independent risk factor of secondary cancer in the multivariable logistic regression analysis.

White patients were more likely to develop secondary cancer (OR = 1.34, 95% CI: 1.03-1.75; *P* = 0.032) setting Asian patients as reference. Patients with stage IV (OR = 0.47, 95% CI: 0.31-0.74; *P* < 0.001), III (OR = 0.53, 95% CI: 0.34-0.82; *P* = 0.004), and II (OR = 0.62, 95% CI: 0.41-0.96; *P* = 0.030) were less likely to have secondary cancer. Patients receiving chemotherapy were more likely to develop secondary cancer (OR = 1.55, *P* = 0.028).

### Development of a prediction nomogram

**Figure 8:**
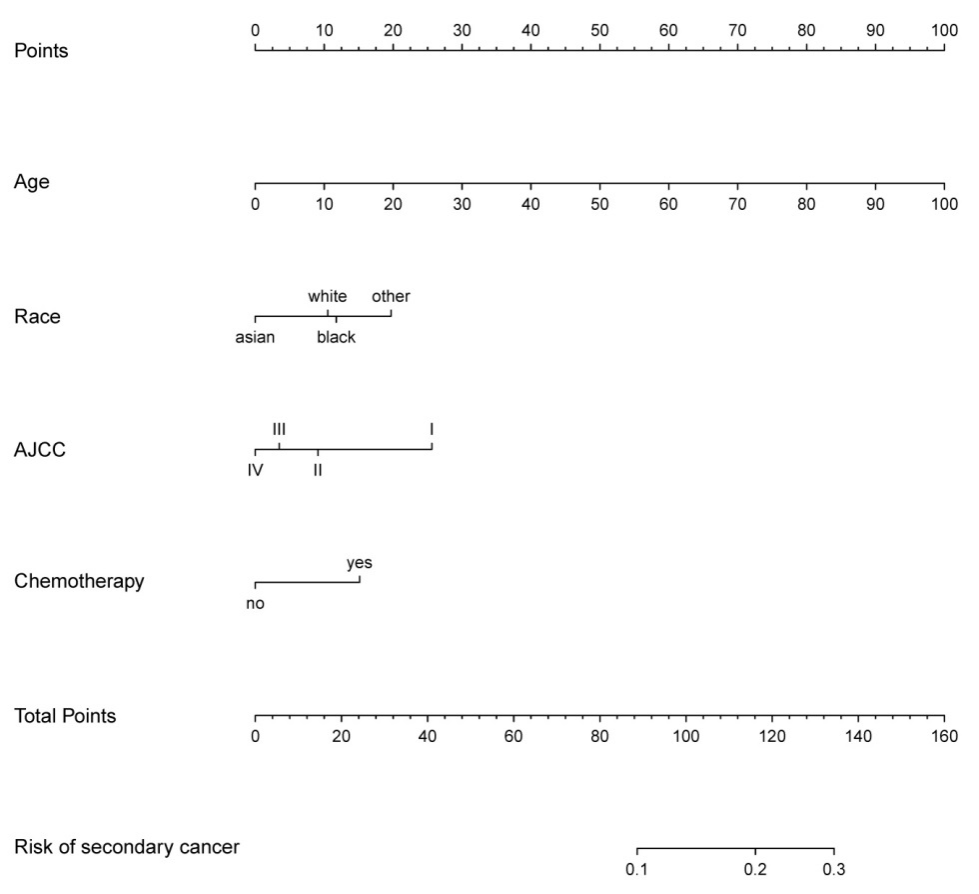
Nomogram of secondary cancer. The nomogram was developed based on the result of the multivariable logistic regression analysis.

The prediction nomogram that incorporated the factors selected in the multivariable logistic regression analysis was developed (Figure 8). The score for each independent risk factor was determined by drawing a line from the factor to the points axis. The sum of the points was located on the total points axis. The probability of development of secondary cancer was located on the points drawing straight down to the risk of secondary cancer axis.

### Prediction of nomogram performance

**Figure 9:**
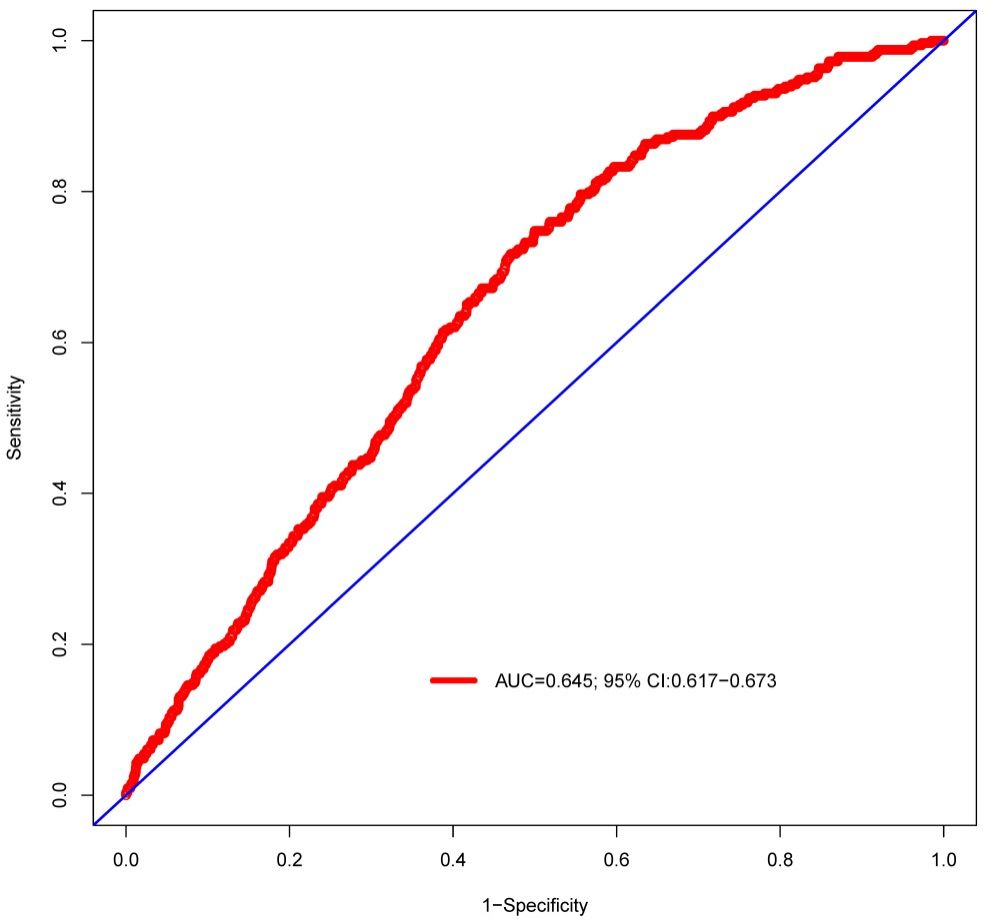
Predictive performance of the nomogram. The AUC is 0.645 (95% CI 0.617-0.673).

**Figure 10:**
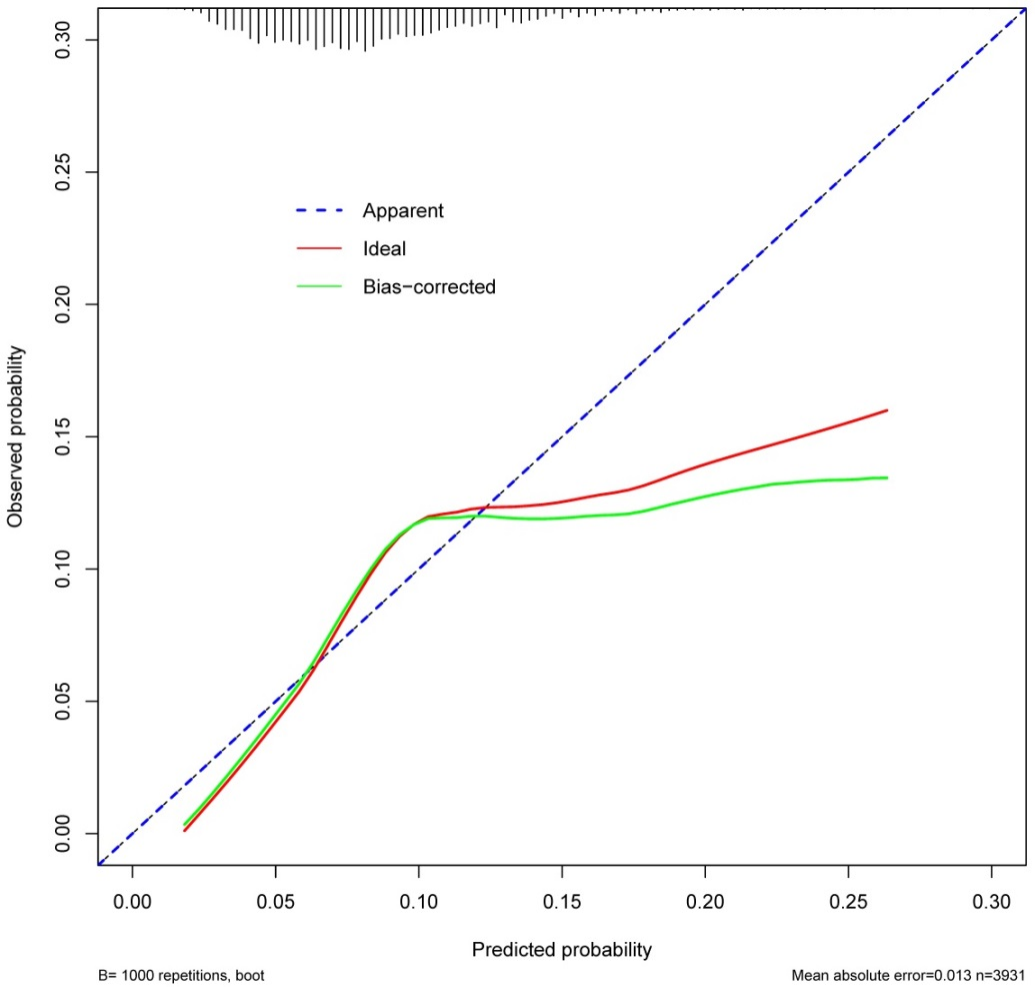
The Calibration curve of the nomogram for predicting secondary cancer. The y-axis represents the actual secondary cancer rate. The x-axis represents the predicted secondary cancer risk. The blue line represents a perfect prediction by an ideal model. The red line represents the performance of the nomogram. The green line represents the performance of the nomogram with bias corrected.

ROC curve was established to assess the accuracy of the nomogram (Figure 9). The AUC of the nomogram was 0.645 with a 95% CI ranging from 0.617 to 0.673. The nomogram was internally validated by computing the bootstrap-corrected Harrell index and by the calibration plot (Figure 10). The calibration plot showed that the probability of secondary cancer predicted by the nomogram was relatively matched.

### Clinical Use

**Figure 11:**
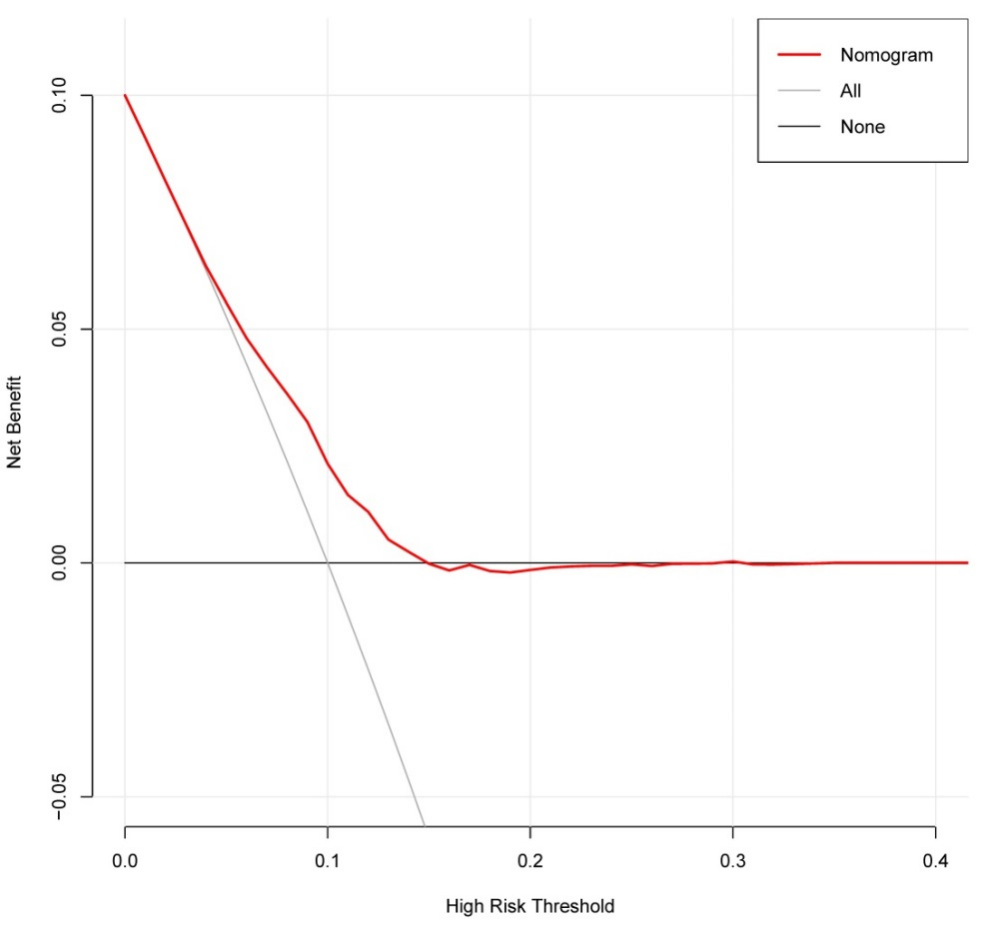
Decision curve analysis for the nomogram. The y-axis measures the net benefit. The grey line represents the assumption that all patients have secondary cancer. The black solid line represents the assumption that no patients have secondary cancer. The red line represents the nomogram.

**Figure 12:**
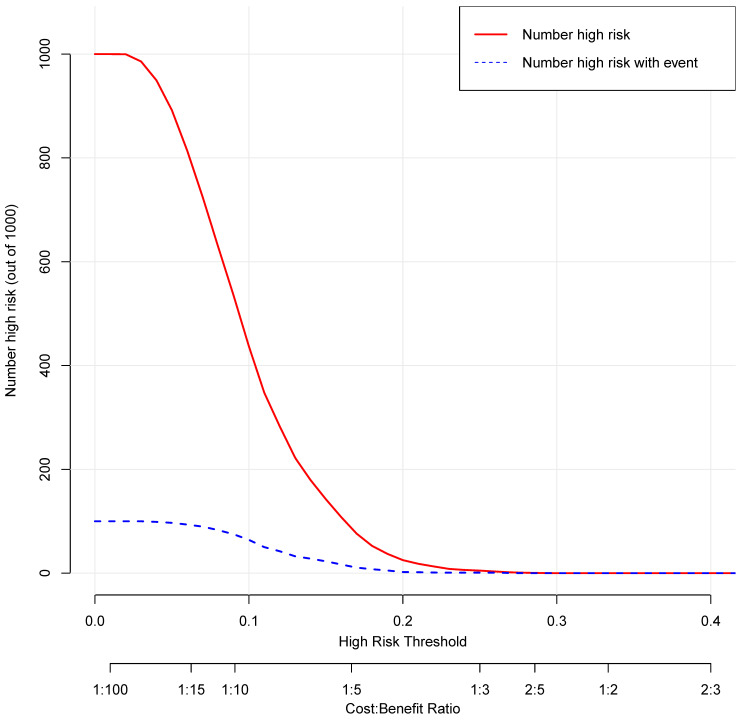
Clinical impact curve for the nomogram. The red curve indicates the number of people who are classified as positive (high risk) by the nomogram at each threshold probability. The blue curve is the number of true positives at each threshold probability.

The decision curve analysis for the nomogram was presented in Figure 11. The decision curve showed that if the threshold probability is between 4% and 25%, using the nomogram added more benefit than either the treat-all-patients scheme or the treat-none scheme. The clinical impact curve for the nomogram was showed in Figure 12.

## Discussion

This retrospective study identified several independent risk factors associated with secondary cancer of NPC after radiotherapy. Although some studies had investigated the risk factor of secondary cancer. Risk factors needs to be further assessed due to the low incidence of secondary cancer 12, 13. The current study investigated the potential risk factors based on a large sample size. Moreover, we established and internally validated a nomogram based on age, race, AJCC, and chemotherapy for predicting secondary cancer. This predictive nomogram could provide personalized estimates of secondary cancer development to guide follow-up strategy for NPC patients. Patients might benefit from this nomogram.

The mechanism of secondary cancer after radiotherapy was not yet clear. Previous studies had showed that the risk factors for secondary cancer included hereditary susceptibility, age of initiative irradiation, the type of primary tumor, the toleration of the irradiated tissues, the dose and area of irradiation, and combination of chemotherapy 14, 15. Our study revealed similar results. White patients were more likely to develop secondary cancer (OR = 1.34, 95% CI: 1.03-1.75; *P* = 0.032) setting Asian patients as reference. Moreover, older age was more likely to develop secondary cancer (OR = 1.03, 95% CI: 1.02-1.04; *P* < 0.001).

The multivariable logistic regression analysis revealed that patients with stage IV (OR = 0.47, 95% CI: 0.31-0.74; P < 0.001), III (OR = 0.53, 95% CI: 0.34-0.82; P = 0.004), and II (OR = 0.62, 95% CI: 0.41-0.96; P = 0.030) were less likely to have secondary cancer. This was an unexpected finding. In clinical practice, patients with locoregionally advanced diseases would receive more chemotherapy compared to early-stage diseases. Based on the multivariable logistic regression of our study, patients with locoregionally advanced diseases were more likely to develop secondary cancers. The possible explanation was that locoregionally advanced diseases had worse survival compared with early-stage diseases. The survival time might be insufficient to develop secondary cancers.

Our study suggested that chemotherapy was not a risk factor of secondary cancer in the univariate logistic regression analysis. However, chemotherapy was an independent risk factors of secondary cancer in the multivariable logistic regression analysis. This was another unexpected finding. It was reported that chemotherapy could increase the incidence of secondary cancer and reduce the latency between radiotherapy and secondary cancer occurrence 13, 16. However, a recently published study revealed that chemotherapy was not an independent risk factor of secondary cancer 17, 18. Until now, the effect of chemotherapy on secondary cancer was still unclear due to the limited studies. Our study with a large sample size found that chemotherapy was associated with the incidence of secondary cancer. The result needed to be verified in prospective studies with longer follow-up time.

Until now, latency of secondary cancer is unclear. It was reported that the latency period for development of secondary cancer was between 3 and 36 years (median: 8.5 years) after radiotherapy 17. On the other hand, the latency of secondary cancer was shorter for patients who received intensity-modulated radiotherapy than that for patients who received conventional radiotherapy (median years: 4.0 vs. 11.0, *P* = 0.013) 11. Due to the limitations of SEER database, the latency of secondary cancer after radiotherapy could not be extracted. Thus, the latency period for development of secondary cancer could not be calculated. However, the overall survival of secondary cancer group was worse than that of non-secondary cancer group in 7^th^ year after radiotherapy. This result might indicate that the latency of secondary cancer was less than 7 years.

This nomogram revealed that the AUC was 0.645 (95% CI: 0.617 to 0.673). The result suggested that the discriminatory capacity of the nomograms was relatively weak. Moreover, calibration curves, used to quantify how close predictions were to the actual outcome, showed that prediction was also not well calibrated. The possible explanations were as following: (1) Secondary cancer of NPC after radiotherapy was rare. The frequency of secondary cancer was very low. Several studies reported that the incidence of secondary cancer ranged from 0.8% to 5.6% 7-10. Although our study reported a percent of 8.37% for secondary cancer after radiotherapy, the sample size of patients with secondary cancer was still small. The small sample size might have been insufficient for establishing a nomogram for prediction of secondary cancer in NPC patients after radiotherapy. (2) Only 4 risk factors of secondary cancer were identified in the multivariable logistic regression analysis. The nomogram was established based on the risk factors of age, race, AJCC, and chemotherapy. Important factors of radiation therapy technique, radiation dose and its distribution were not included due to the limitation of SEER database 19-21. Thus, the nomogram could not provide well-discriminating ability. This nomogram should be modified with more independent risk factors to improve its efficacy.

Limitations of this study should be considered. First, considering the low incidence of secondary cancer, the nomogram was only internally validated by computing the bootstrap-corrected Harrell index and by the calibration plot. The nomogram was not externally verified in a validation cohort. Its clinical utility should be treated with caution. Second, the locations of secondary cancer were not provided in the SEER database. It was unclear whether the secondary cancer was more likely to occur in the fields of radiotherapy.

In conclusion, age, race, AJCC stage, and chemotherapy were independent risk factors of secondary cancer in nasopharyngeal carcinoma patients after radiotherapy. Multicenter studies with large sample sizes and longer follow-up time are needed to verify the nomogram of this study.

## Figures and Tables

**Table 1:** Patient characteristics.

	Non-secondary cancer (n=3602)	Secondary cancer (n=329)	*P*
Age (year)			<0.001
median	59	53	
IQR	52-65	43-63	
Race			0.008
asian	1486 (41.3%)	106 (32.1%)	
black	422 (11.7%)	39 (11.9%)	
white	1614 (44.8%)	173 (52.6%)	
other	80 (2.22%)	11 (3.34%)	
Sex			0.164
female	1102 (30.6%)	88 (26.7%)	
male	2500 (69.4%)	241 (73.3%)	
Grade			0.174
I	56 (1.5%)	9 (2.8%)	
II	280 (7.7%)	35 (10.6%)	
III	1119 (31.1%)	101 (30.7%)	
IV	1133 (31.5%)	96 (29.2%)	
unknown	1014 (28.2%)	88 (26.7%)	
Pathology			0.192
WHO I	1124 (31.2%)	121 (36.8%)	
WHO II	881 (24.5%)	70 (21.3%)	
WHO III	709 (19.7%)	59 (17.9%)	
other	888 (24.6%)	79 (24.0%)	
T stage			0.096
1	1148 (31.9%)	121 (36.8%)	
2	927 (25.7%)	91 (27.7%)	
3	739 (20.5%)	60 (18.2%)	
4	788 (21.9%)	57 (17.3%)	
N stage			0.042
0	867 (24.1%)	101 (30.7%)	
1	1218 (33.8%)	109 (33.1%)	
2	1078 (29.9%)	87 (26.4%)	
3	439 (12.2%)	32 (9.8%)	
AJCC			0.007
I	296 (8.3%)	42 (12.8%)	
II	916 (25.4%)	95 (28.9%)	
III	1230 (34.1%)	104 (31.6%)	
IV	1160 (32.2%)	88 (26.7%)	
Chemotherapy			0.897
no	445 (12.4%)	42 (12.8%)	
yes	3157 (87.6%)	287 (87.2%)	

IQR: interquartile range. WHO: World Health Organization. AJCC: American Joint Committee on Cancer.

**Table 2:** Patient characteristics after propensity score matching.

	Non-secondary cancer (n=322)	Secondary cancer (n=322)	*P*
Age (year)			0.672
median	59	58	
IQR	51-67	52-65	
Race			0.751
asian	116 (36.0%)	106 (32.9%)	
black	30 (9.3%)	37 (11.5%)	
white	10 (3.1%)	10 (3.1%)	
other	166 (51.6%)	169 (52.5%)	
Sex			0.860
female	90 (28.0%)	87 (27.0%)	
male	232 (72.0%)	235 (73.0%)	
Grade			0.741
I	7 (2.2%)	8 (2.4%)	
II	32 (10.0%)	34 (10.6%)	
III	96 (29.8%)	100 (31.1%)	
IV	108 (33.5%)	92 (28.6%)	
unknown	79 (24.5%)	88 (27.3%)	
Pathology			0.345
WHO I	65 (20.2%)	74 (23.0%)	
WHO II	108 (33.5%)	119 (37.0%)	
WHO III	73 (22.7%)	70 (21.7%)	
other	76 (23.6%)	59 (18.3%)	
T stage			0.991
1	114 (35.4%)	114 (35.4%)	
2	89 (27.6%)	91 (28.3%)	
3	59 (18.4%)	60 (18.6%)	
4	60 (18.6%)	57 (17.7%)	
N stage			0.489
0	85 (26.4%)	94 (29.2%)	
1	128 (39.8%)	109 (33.9%)	
2	79 (24.5%)	87 (27.0%)	
3	30 (9.3%)	32 (9.9%)	
AJCC			0.326
I	23 (7.1%)	35 (10.9%)	
II	109 (33.9%)	95 (29.5%)	
III	102 (31.7%)	104 (32.3%)	
IV	88 (27.3%)	88 (27.3%)	
Chemotherapy			0.091
no	26 (8.1%)	40 (12.4%)	
yes	296 (91.9%)	282 (87.6%)	

IQR: interquartile range. WHO: World Health Organization. AJCC: American Joint Committee on Cancer.
